# The value of ^18^F-FDG PET/CT and ^18^F-DOPA PET/CT in determining the initial surgical strategy of patients with medullary thyroid cancer: Preoperative PET/CT imaging for medullary thyroid cancer

**DOI:** 10.1186/s40644-025-00862-4

**Published:** 2025-03-26

**Authors:** Eline C. Jager, Adrienne H. Brouwers, Madelon J. H. Metman, Dilay Aykan, Lisa H. de Vries, Lutske Lodewijk, Menno R. Vriens, Schelto Kruijff, Thera P. Links

**Affiliations:** 1 Department of Surgery, Division of Surgical Oncology, University Medical Center Groningen, Groningen, The Netherlands; 2 Department of Internal Medicine, Division of Endocrinology, University Medical Center Groningen, Groningen, The Netherlands; 3 Department of Nuclear Medicine and Molecular Imaging, University of Groningen, University Medical Center Groningen, Groningen, The Netherlands; 4 Department of Surgical Oncology, University Medical Center Utrecht, Utrecht, The Netherlands; 5 Department of Molecular Medicine and Surgery, Karolinska Institutet, Stockholm, Sweden

**Keywords:** Preoperative imaging, PET/CT, Surgery, Lymph node dissection, Medullary thyroid cancer, Deescalation

## Abstract

**Background:**

While total thyroidectomy with central neck dissection (CND) is standard for medullary thyroid cancer (MTC), performing a lateral neck dissection (LND) depends on locoregional metastatic spread and is usually decided per individual. This study evaluated the utility of preoperative PET/CT in staging patients at diagnosis and guiding the initial surgical plan, while also exploring the value of neck ultrasound, MRI, and CT.

**Methods:**

All MTC patients from two tertiary hospitals (2000 – 2020) were identified from two retrospective databases. All reports of neck ultrasounds, MRIs, CTs and PET/CTs < 8 months prior to primary surgery or < 4 months after MTC diagnosis were reviewed. The sensitivity and specificity of each imaging modality for locating locoregional lymph node metastases (LNM) was determined.

**Results:**

A total of 175 MTC patients were included (91 females and 57 hereditary MTCs). Median age at presentation was 52 years (IQR 38 – 62). Initial treatment included a total thyroidectomy, CND and LND in 155 (89%), 140 (80%) and 59 (33%) patients. Preoperative imaging of the neck included ultrasound (91, 52%), MRI (33, 19%) and CT (31, 18%). PET/CT imaging was performed in 56 (32%) patients (35 ^18^F-FDG PET/CTs and 33 ^18^F-DOPA PET/CTs). Sensitivity for LNM in the central compartment was 72%, 39%, 6%, 42% and 93% for ^18^F-FDG PET/CT, ^18^F-DOPA PET/CT, ultrasound, MRI and CT, respectively. Respective specificity rates were 80%, 100%, 100%, 71% and 100%. Sensitivity rates for lateral neck LNM were 89%, 81%, 77%, 76% and 75%, for ^18^F-FDG PET/CT, ^18^F-DOPA PET/CT, ultrasound, MRI and CT, while specificity rates were 100%, 100%, 75%, 78% and 50%, respectively. Twenty-three patients had distant metastases on imaging. In total, 14 ^18^F-FDG PET/CTs and 9 ^18^F-DOPA PET/CTs were made in these 23 patients (both in six patients). All but one PET/CT showed distant metastases.

**Conclusions:**

PET/CT is a powerful tool to detect locoregional LNM and can particularly help identify cases where LNDs are required, avoiding reoperation later on. For accurate staging of the central neck, PET may be combined with diagnostic CT. Finally, PET/CT’s ability to detect distant metastases may support de-escalation of a surgical intervention when cure is unlikely.

**Supplementary Information:**

The online version contains supplementary material available at 10.1186/s40644-025-00862-4.

## Introduction

The only way to reach complete curation in patients with localized medullary thyroid carcinoma (MTC) is through comprehensive high-quality surgery with removal of the affected thyroid and lymph node metastases (LNM). Treatment guidelines recommend a total thyroidectomy in all patients since both thyroid lobes can be affected, particularly in patients with hereditary MTC [1].

However, there is no consensus on the optimal extent of the initial lymph node neck dissection. Due to high prevalence of LNM in the central compartment (level VI), a prophylactic central neck dissection (CND) is considered the standard of treatment and often performed directly following total thyroidectomy [2]. Removal of the lymph nodes (LNs) in the lateral neck (levels II-V) is mostly subject of discussion when designing a treatment plan. Some expert centers recommend an aggressive surgical approach including standard dissection of all compartments, to induce an as low as possible tumor burden and low tumor markers, hoping to reduce any risk of local recurrence [3]. However, aggressive routine removal of all lymph nodes may also result in negative findings and little survival gain, whilst risking significant morbidity with no guarantee to avoid future recurrences [4–6]. Also, recurrence in an extensively operated area, is associated with higher risks and surgical difficulty when the need to reoperate arises.

The American Thyroid Association (ATA) guidelines of 2015 suggest a lateral neck dissection (LND) when preoperative imaging indicates LNM [1]. Ultrasonography is advised in all patients undergoing thyroid surgery, with a sensitivity and specificity to detect lateral LNM in 56% and 97% of cases, respectively [7]. CT and MR are not standard in the diagnostic work-up but may be performed to provide anatomical information to guide the surgical approach, especially in extensive locoregional disease [8].

There is no recommendation to perform preoperative PET/CT imaging in the ATA or European Association of Nuclear Medicine (EANM) guidelines, nor is there diagnostic evidence pointing to a particular PET tracer in this setting. As a result, many centers have developed their own guidelines for preoperative PET/CT imaging—including the choice for a specific PET tracer—and subsequent extent of initial surgery. During follow-up, ^18^F-DOPA PET/CT is generally agreed to be the best for detecting recurrent disease [9], while ^18^F-FDG PET/CT is valuable when dedifferentiation is suspected [10]. ^18^F-FDG avidity is a result of the superior glucose metabolism, while ^18^F-DOPA is taken up by cells with high AADC enzymatic activity, as seen in tumors with neuro-endocrine origin [11, 12].

To explore the value of PET/CT in the preoperative setting, we conducted the current observational study. The study aims to describe the yield of preoperative ^18^F-FDG and/or ^18^F-DOPA PET/CT imaging in patients undergoing surgery for MTC, with a particular focus on the lateral neck compartment. In addition, the value of PET/CT in patients with distant metastases was explored.

## Method

### Patients

This retrospective, observational study evaluated preoperative imaging and extent of initial surgery of MTC patients from two large tertiary referral centers in the Netherlands. In patients not undergoing surgery, initial imaging at diagnosis was also explored. Pseudonymized data from all MTC patients diagnosed between 2000—2020 were extracted from databases of the University Medical Center of Groningen (UMCG) and the University Medical Center of Utrecht (UMCU). Patients with incidentally diagnosed MTCs upon histopathological examination and patients with lacking data on preoperative imaging or treatment were excluded.

### Preoperative PET/CT imaging

All preoperative ^18^F-FDG and ^18^F-DOPA PET/CTs < 8 months prior to initial thyroid surgery were identified. In patients not undergoing surgical treatment, any PET/CT performed in the initial diagnostic work-up was identified (< 4 months after cyto- or histological MTC diagnosis). Their reports were critically reviewed and variables on the location of ^18^F-FDG or ^18^F-DOPA pathological tracer uptake in the thyroid, locoregional LNs and distant locations were extracted. ^18^F-FDG or ^18^F-DOPA tracer uptake in LNs was categorized as being in the central compartment (level VI) or the right or left lateral compartment (levels II – IV left and right).

### Conventional imaging

Any neck CT, MRI or ultrasound < 8 months prior to primary thyroid surgery (operated patients) or < 4 months after first cyto- or histological MTC diagnosis (non-operated patients) was identified. Reports of all imaging modalities were reviewed in detail to determine the location of the suspected primary thyroid tumor and cervical lymphadenopathy (central, right/left lateral compartment), as reported by expert head-neck radiologists.

### Imaging definitions

For patients undergoing total thyroidectomy, preoperative imaging was considered true positive when the malignant tumor (or tumors in case of bilateral disease) was identified in the correct lobe. Imaging was only considered true negative when imaging did not show any thyroid abnormality and histopathology confirmed absence of a tumor in both lobes. False negatives had positive histopathology but were negative on preoperative imaging. Imaging was false positive when a thyroid abnormality was identified, without confirmation on histopathology.

For LNM in the central neck (one compartment per patient) and lateral neck (two compartments per patient), true/false positive and negatives rates were also determined for the patients undergoing CND or LND, per imaging modality. Imaging was true positive if abnormal LNs were found on both imaging and corresponding histopathology, and true negative if no abnormalities were found on either. False positives occurred when imaging showed lymphadenopathy, but histopathology did not confirm it, while false negative had normal imaging but positive histopathology.

Patients were considered to have distant metastases (M1) when identified on imaging (preoperatively or < 4 months after diagnosis), all other patients were classified as M0/MX. Metabolically active lesions exhibiting activity visually above physiological uptake in anatomical sites distant from the primary tumor were defined as M1 for PET/CT. In a few instances, patients were identified with distant metastases based on diagnostic (chest) CT or MRI of the spinal column.

### Surgery and histopathology

Extent of surgery was determined from surgery and histopathology reports. The location of the primary tumor in the thyroid was determined (left, right, bilateral) and the presence of metastatic LNs in the central and lateral (left, right, bilateral) neck compartments were determined. No distinction between right- or left-sided CND was made due to standard dissection of both sides and no clear discrimination in surgical reports. If a lymph node dissection was clearly reported in the surgical report, without description of the resected LNs in the histopathology report, the LNs were assumed to be unaffected.

### Calcitonin measurements

Preoperative calcitonin measurements were available in a subset of patients. Due to the use of five different calcitonin assays over time, analysis of their absolute values was impossible and values were categorized into ranges: < 500 and ≥ 500 ng/L. Patients with calcitonin < 10 ng/L one year after primary thyroid surgery were considered biochemically cured.

### Definitions

Patients were classified according to the 8th edition of the Tumor-Node-Metastasis system by the American Joint Cancer Committee (AJCC) and staged as I-IV based on categories defined by the AJCC [13]. MTCs were considered sporadic in the absence of a germline *RET* mutation, or absence of Multiple Endocrine Neoplasia type 2 (MEN2)-related diseases and family history when no germline analysis was performed. Overall survival (OS) and disease-specific survival (DSS) were based on a patient’s status at last available follow-up at the time of data collection. All surgeries for recurrent or persistent disease more than 1 year after diagnosis were considered locoregional recurrences. Decade 1 refers to the period from January 2000 to June 2010, while Decade 2 spanned from July 2010 to December 2020.

### Statistical analysis

Descriptive statistics report characteristics; medians and inter-quartile ranges (IQR) for continuous (non-normally distributed) data and counts (percentages) for categorical data. Differences between groups were determined by Fisher’s Exact Test (categorical data) or Mann-Whitney U (continuous data). True positive, false positive, true negative and false negatives were determined per imaging modality in relation to available histopathology of the thyroid, central neck compartment and lateral neck compartments. For CND and LND, two-way contingency tables were constructed to calculate the sensitivity, specificity, positive predictive value, negative predictive value and accuracy of ^18^F-FDG PET/CT, ^18^F-DOPA PET/CT and conventional imaging, respectively (compartment-based assessment). All analyses were performed in IBM SPSS Statistics 23 for Windows. *P*-values < 0.05 were considered statistically significant.

### Ethics

The Institutional Review Boards of the UMCG and UMCU approved the study (UMCG-10779, UMCU-20–393). All study procedures were conducted in accordance with the Helsinki Declaration.

## Results

### All patients

**Fig. 1:**
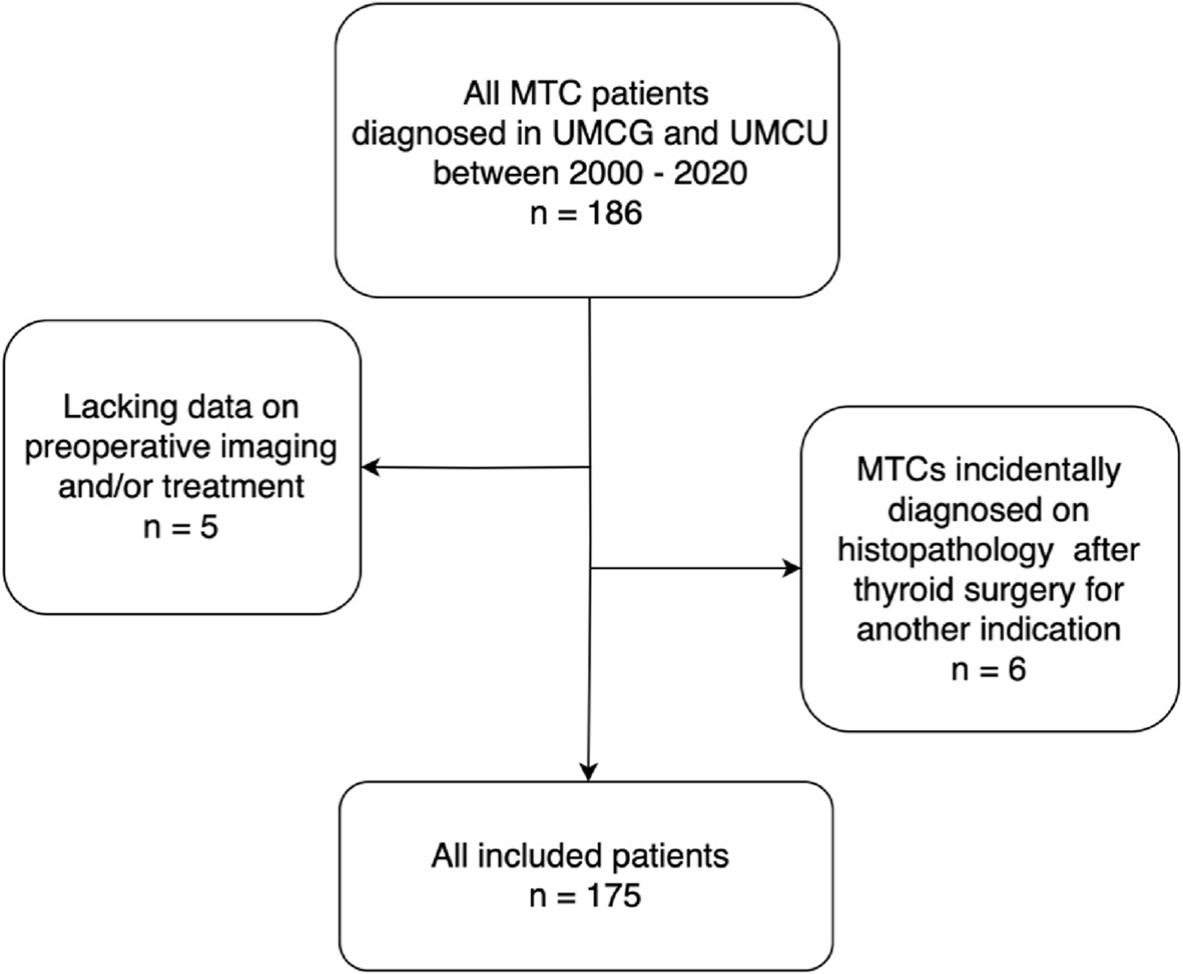
Flow diagram displaying patient selection

A total of 186 MTC patients were diagnosed between January 2000 and December 2020 in the UMCG and UMCU. After applying exclusion criteria (see Fig. 1), the total cohort consisted of 175 patients. Of these patients, 91 (52%) were female and 57 (33%) had hereditary MTC. The median age at presentation was 52 years (IQR 38 – 62) and 159 (91%) underwent surgical treatment. The other 16 patients were treated in a palliative setting due to extensive local disease or distant metastases (tyrosine kinase inhibitors [TKI] 5 patients, locoregional radiotherapy 6 patients, no treatment 5 patients). At final follow-up 36 (21%) patients had died; 22 (13%) died due to MTC disease progression. Additional patient and treatment characteristics can be found in Table 1.

**Table 1:** Patient and treatment characteristics

	All patients *n*=175 N (%) or median (IQR)
Academic hospital	
UMCG	101 (58)
UMCU	74 (42)
Age in years (median)	52 (38 – 62)
Sex	
Male	84 (48)
Female	91 (52)
Type of MTC	
Sporadic	118 (67)
Familial	57 (33)
T-stage	
TX	9 (5)
T1-2	122 (70)
T3-4	44 (25)
N-stage	
N0	76 (43)
N1a	33 (19)
N1b	66 (38)
M-stage	
MX/M0	152 (87)
M1	23 (13)
TNM category	
I - II	76 (43)
III – IV	99 (57)
Calcitonin^a^	
<500	44 (32)
≥500	95 (68)
Thyroid surgery	159 (91)
Total thyroidectomy	155 (89)
Hemithyroidectomy	4 (2)
Lymph node dissection	144 (82)
Central neck dissection	140 (80)
Lateral neck dissection	59 (33)
Bilateral tumors^b^	46/155 (30)
Lymph node metastases	
Central neck (level VI)	71/140 (51)
Lateral neck (level II-V)	53/59 (90)
Follow-up time (months)	65 (29 – 125)
Locoregional recurrence	21 (12)
Time to locoregional recurrence (months)	29 (17 – 51)
Biochemical cure 1-year after diagnosis^c^	86 (49)
Disease-specific survival	153 (87)
Overall survival	139 (79)

*Abbreviations*: *MTC *Medullary thyroid cancer

^a^Available in 139/175 patients

^b^Bilateral tumors in patients undergoing total thyroidectomy (9/46 sporadic and 37/46 hereditary cases, *p*<0.001). In 4/155 patients, the laterality of the tumor could not be determined and in 1/155 patients, only extrathyroidal MTC localizations were identified

^c^Unknown for 14 patients, percentages and significance of total excluding unknown variables are given. The majority of the hereditary MTCs and TNM stage I-II patients were biochemically cured after 1 year (41/54 hereditary MTC patients *p*<0.001; 69/72 TNM stage I-II patients, *p*<0.001)

### Preoperative imaging

Preoperative imaging of the neck was performed in 133 (76%) of 175 patients, most (94/133, 71%) was performed in decade 2 (*p* < 0.001). Preoperative imaging consisted of one, two, three or four modalities in 68 (39%), 35 (20%), 25 (14%) and 5 (3%) patients. Neck ultrasound was performed in 91 (52%) of all patients. Neck CT (31, 18%) and MRI (33, 19%) were less prevalent. A PET/CT was performed in 56 patients (32%), 42/56 (75%) took place in the second decade (*p* = 0.012). Of 175 patients, 77 (44%) patients had conventional imaging only (MRI, CT and/or ultrasound), 44 (25%) had conventional imaging as well as PET/CT imaging, and in 12 patients (7%) PET/CT was the only preoperative imaging modality. Of the 42 patients without preoperative imaging of the neck, 25 (60%) had hereditary MTC (*p* < 0.001).

## Preoperative PET/CT imaging

**Fig. 2.**
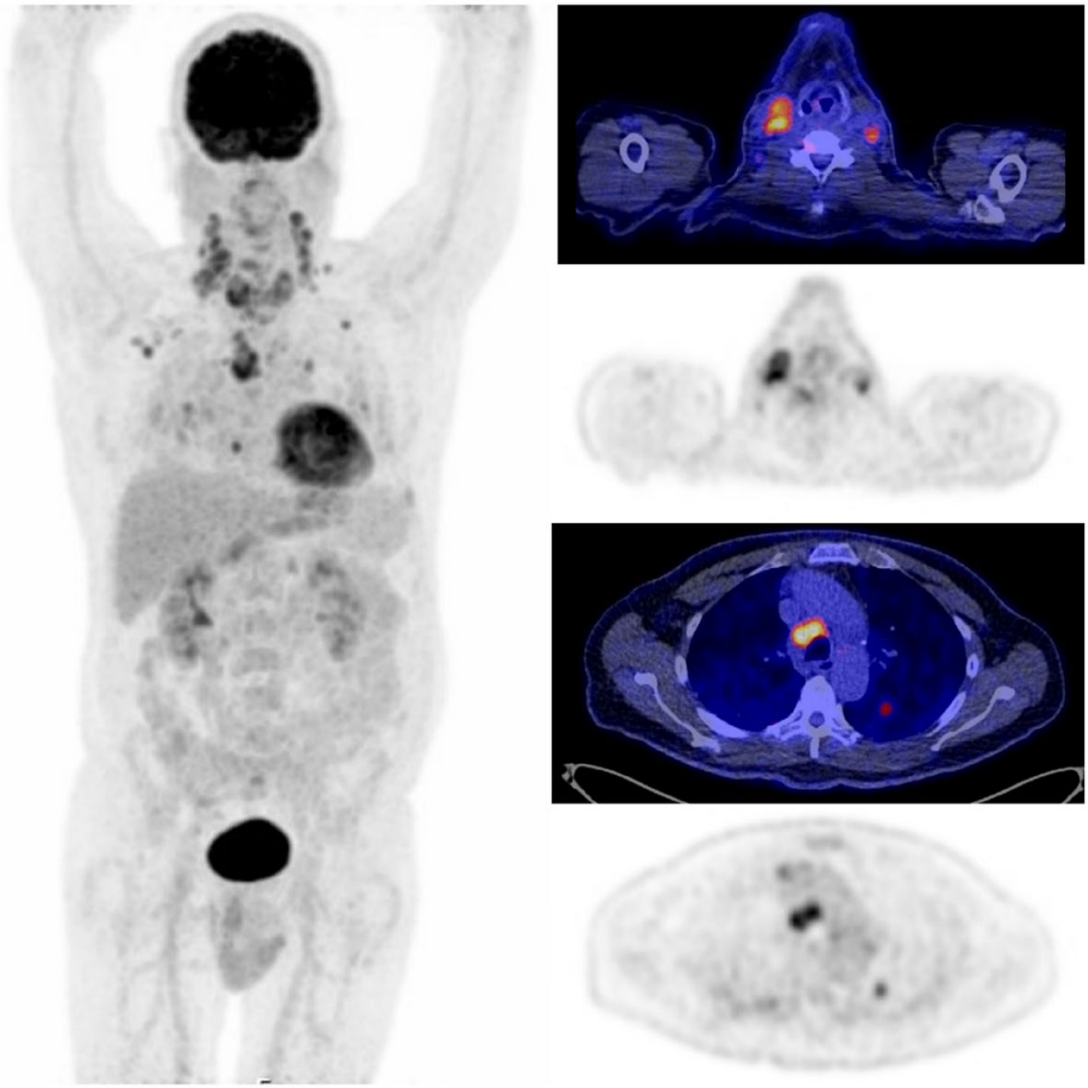
^18^F-FDG PET/CT of a patient with extensive bilateral lymph node metastases in the neck and distant metastases in lungs

**Fig. 3:**
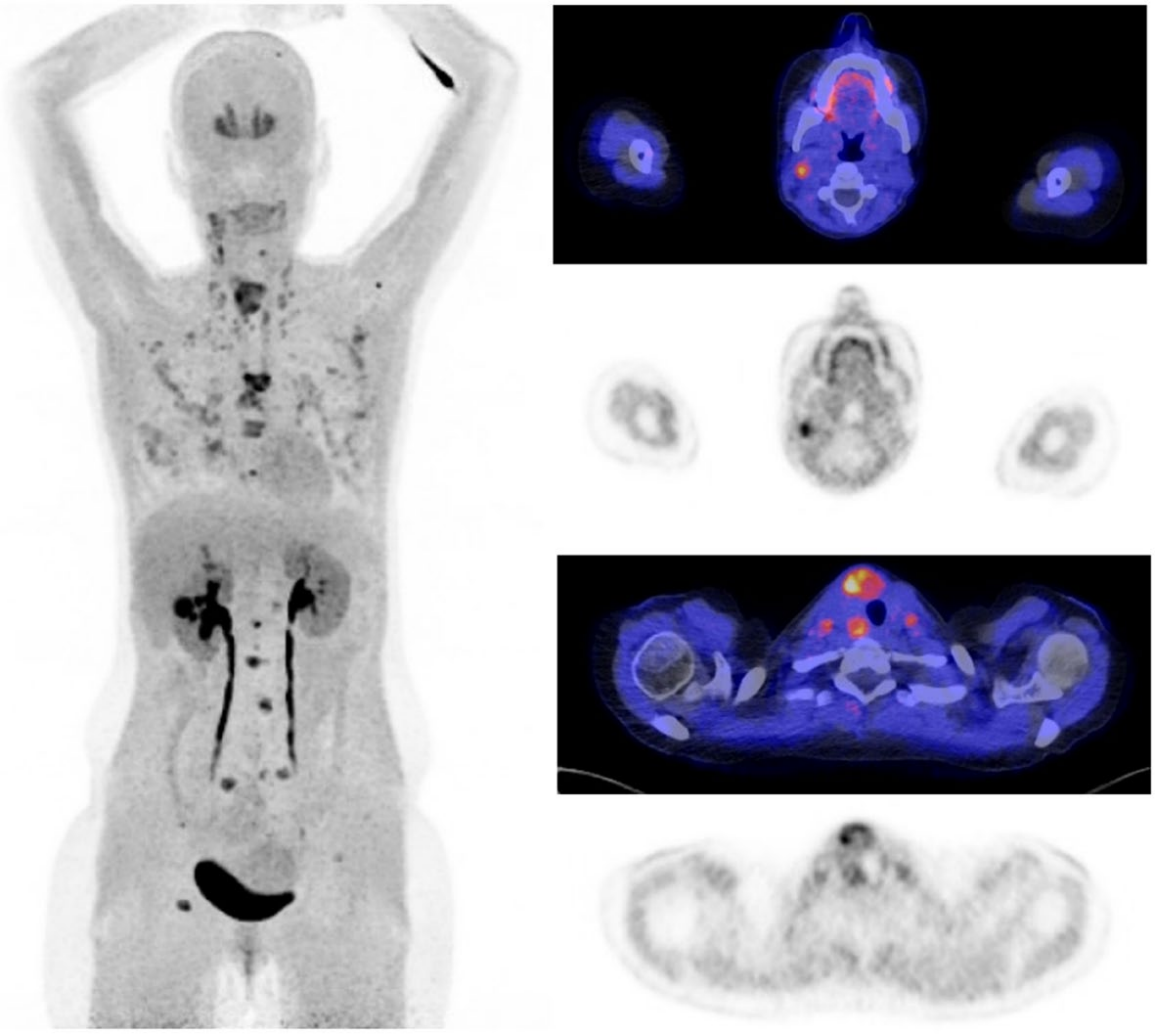
Images of an ^18^F-DOPA PET/CT performed preoperatively in a patient with medullary thyroid cancer with locoregional lymph node metastases. In addition, the ^18^F-DOPA PET/CT identified distant metastatic lesions in both lungs and bone

In total, 35 ^18^F-FDG PET/CTs and 33 ^18^F-DOPA PET/CTs were undertaken. In 12 patients both scans were performed. PET/CTs were mainly performed in the UMCG (48 patients vs 8 patients in UMCU, *p* < 0.001). ^18^F-DOPA PET/CT was not available in the UMCU. PET/CT identified 18 of 23 patients with distant metastatic disease. Images of two patients in whom an ^18^F-FDG PET/CT or ^18^F-DOPA PET/CT was conducted are shown in Figs. 2 and 3, respectively.

The most common indication for a PET/CT was to assess disease extent in suspected or confirmed MTC, occurring in 46/56 (82%) patients. Another reason was pheochromocytoma screening in MEN2A patients (7/56, 13%), all performed with DOPA PET/CTs. In 3/56 (5%), PET/CT was done for cervical lymphadenopathy without a known primary tumor (2/56) or for other malignancies (1/56), all leading to MTC diagnosis.

Patients with a preoperative calcitonin of ≥ 500 ng/l were more likely to have preoperative PET/CT imaging than those with calcitonin < 500 ng/l (45% versus 16%, *p* < 0.001). Locoregional recurrences occurred in 5 (9%) and 16 (13%) of patients with and without preoperative PET/CT, respectively (*p* = 0.534). Patients with PET/CT had worse disease-specific survival (HR 8.319, *p* = 0.004) and overall survival (HR 10.323, *p* = 0.001) compared to those without preoperative PET/CT. This difference disappeared when excluding patients with distant metastasis (HR 0.001 *p* = 0.975 and HR 0.767 *p* = 0.381, respectively).

## ^18^F-FDG PET/CT

Of 35 patients with an ^18^F-FDG PET/CT, 25 were surgically treated (24 total thyroidectomy, 1 palliative hemithyroidectomy). These 25 ^18^F-FDG PET/CT were made 63 days (IQR 48 – 95) prior to surgery. Preoperative calcitonin (available in 30 patients) was < 500 in 2 (7%) and ≥ 500 in 28 (93%) patients.

### Primary tumor

^18^F-FDG PET/CT showed increased ^18^F-FDG uptake in the thyroid gland in 30/35 (86%) patients (bilateral uptake in 3 patients). Subsequent total thyroidectomy in 24/35 patients confirmed intrathyroidal MTC in 23/24 patients (median tumor size 25 mm, IQR 13 – 42). In one patient, only extrathyroidal localizations of MTC were identified (true negative on ^18^F-FDG PET/CT). The ^18^F-FDG PET/CT identified the malignant tumor(s) in the correct lobe (true positive) in 17/24 (71%) cases (tumor size: 31 mm, IQR 20 – 46). In 6/24 patients (25%) (of which 3/6 bilateral thyroid tumors), the MTC tumor was not identified in the correct lobe or lobes (false negative). These tumors (10 mm, IQR 9 – 23) were smaller than true positive tumors (*p* = 0.019).

### Central compartment lymph nodes

Of 35 evaluated central compartments on ^18^F-FDG PET/CTs, ^18^F-FDG uptake was identified in 23/35 (66%) central compartments. CND was performed in 23/35 patients and 18/23 (78%) were positive for LNM on histopathology (see Table 2, Fig. 2). Thirteen of these 18 central compartments were correctly identified on the preoperative ^18^F-FDG PET/CT (sensitivity 72%). Of five negative compartments on histopathology, four were correctly identified as negative on ^18^F-FDG PET/CT (specificity 80%).

**Table 2:** Detection of primary thyroid tumor and positive central and lateral compartments on ^18^F-FDG PET/CT, ^18^F-DOPA PET/CT and conventional imaging, respectively

	^18^F-FDG PET/CT	^18^F-DOPA PET/CT	US^a^	MRI	CT^b^
No. of evaluated thyroid glands	35 (100)	33 (100)	81 (100)	33 (100)	30 (100)
No. of total thyroidectomies	24 (69)	26 (79)	74 (91)	25 (76)	19 (63)
True positive, n	17	15	51	12	11
True negative, n	1	0	0	1	2
False positive, n	0	0	17	3	1
False negative, n	6	11	6	9	5
No. of evaluated central compartments	35 (100)	33 (100)	64 (100)	33 (100)	30 (100)
No. of operated central compartments^c^	23 (66)	27 (82)	58 (91)	26 (92)	18 (63)
True positive, n	13	7	2	8	13
True negative, n	4	9	23	5	4
False positive, n	1	0	0	2	0
False negative, n	5	11	33	11	1
Sensitivity %	72%	39%	6%	42%	93%
Specificity %	80%	100%	100%	71%	100%
PPV %	93%	100%	100%	80%	100%
NPV %	44%	45%	41%	31%	80%
Accuracy %	74%	59%	43%	50%	94%
No. of evaluated lateral compartments	70 (100)	66 (100)	128 (100)	66 (100)	60 (100)
No. of operated lateral compartments	22 (31)	19 (29)	34 (27)	30 (45)	22 (37)
True positive, n	17	13	23	16	15
True negative, n	3	3	3	7	1
False positive, n	0	0	1	2	1
False negative, n	2	3	7	5	5
Sensitivity %	89%	81%	77%	76%	75%
Specificity %	100%	100%	75%	78%	50%
PPV %	100%	100%	96%	89%	94%
NPV %	60%	50%	30%	58%	17%
Accuracy %	91%	84%	76%	77%	73%

*Abbreviations*: *PPV *Positive predictive value, *NPV *Negative predictive value, *US *Ultrasound

^a^Of 91 patients with a preoperative ultrasound, the thyroid and lymph node compartments were properly assessed in 81 and 64 patients, respectively

^b^30 out of 31 patients with preoperative CT had a report available for analysis

^c^One patient had a palliative hemithyroidectomy with central compartment dissection, explaining why number of total thyroidectomies and central compartment dissection do not necessarily match. Sensitivity = true positive / (true positive + false negative). Specificity = true negative / (true negative + false positive). PPV = true positive / (true positive + false positive). NPP = true negative / (true negative + false negative. Accuracy = (true positive + true negative) / (true positive + true negative + false positive + false negative)

### Lateral compartment lymph nodes

^18^F-FDG PET/CT assessed 70 lateral compartments, 22/70 were operated on and 19 (86%) of these were positive on histopathology. Seventeen of 22 lateral compartments were correctly identified as positive for LNM on ^18^F-FDG PET/CT, providing a sensitivity of 89% (see Table 2, also for negative predictive value, positive predictive value and accuracy). There was no false positive uptake in the lateral compartment (specificity 100%, see Supplementary 1).

## ^18^F-DOPA PET/CT

Twenty-seven of 33 ^18^F-DOPA PET/CTs were performed 69 days (IQR 35 – 160) prior to surgery (26 total thyroidectomy, 1 hemithyroidectomy). Calcitonin (available in 32) was < 500 in 5 patients (16%) and ≥ 500 in 27 (84%). Calcitonin < 500 ng/L was more prevalent among patients with hereditary MTC than those with sporadic disease (63% vs 0%, *p* < 0.001).

### Primary tumor

Pathological ^18^F-DOPA uptake in the thyroid gland was detected in 26/33 (79%) patients (bilaterally in three patients). Histopathology confirmed MTC in all 26 patients undergoing total thyroidectomy (tumor size 19 mm, IQR 10 – 39). The ^18^F-DOPA PET/CT was true positive in 15/26 (58%) patients (tumor size 30 mm, IQR 20 – 50). Malignant tumor(s) was/were not identified in the correct lobe in 11/26 (tumor size 10 mm, IQR 3 – 21), 7 of these 11 had bilateral tumors. False negative tumors were smaller (*p* = 0.001).

### Central compartment lymph nodes

Abnormal ^18^F-DOPA accumulation was seen in 11 central compartments of 33 ^18^F-DOPA PET/CTs (33%). After CND in 27/33 patients, 18 (67%) central compartments were positive for LNM, seven of these were true positive on ^18^F-DOPA PET/CT (sensitivity 39%) (Table 2, Fig. 3). There was no false positive ^18^F-DOPA uptake, specificity was 100%.

### Lateral compartment lymph nodes

Of 66 evaluated lateral neck compartments on ^18^F-DOPA PET/CT, 19 were operated on during surgery confirming LNMs in 16/19 (84%) lateral compartments (Table 2, Supplementary 2). ^18^F-DOPA PET/CT identified 16 lateral compartments correctly (true positives and true negatives) and missed three (false negatives), giving a sensitivity and specificity of 81% and 100%, respectively.

## Conventional imaging

Neck ultrasound, MRI and CT were performed in 91, 33 and 31 patients, respectively (reports were available in 85/91, 33/33 and 30/31 patients, respectively). Ultrasound reports included information on the thyroid in 81/91 patients and on lymph nodes in 64/91 cases. Correct identification of the primary thyroid tumor(s) (true positive and true negatives) was seen in 69%, 52% and 68% of neck ultrasounds, MRIs and CTs (see Table 2). Sensitivity rates for LNMs in the central compartment varied widely (6% ultrasound, 42% MRI and 93% CT) while specificity rates were generally high (100% ultrasound, 71% MRI and 100% CT). Concerning LNMs in the lateral compartments, sensitivity rates were comparable (ultrasound 77%, MRI 76%, CT 75%). Specificity rates were 75%, 78% and 50%, respectively.

## Distant metastases

A total of 23 patients were identified with distant metastatic disease on preoperative imaging. Eleven were first identified with ^18^F-FDG PET/CT or ^18^F-DOPA PET/CT. Another 11/23 were identified with conventional imaging, extending beyond the neck. One patient presented with impending spinal cord injury due to a metastasis in the spinal column, which led to the diagnosis of M1 disease. In total, 23 PET/CTs were made in this cohort of patients, 14 ^18^F-FDG PET/CTs and 9 ^18^F-DOPA PET/CTs (both scans in six patients). Except for one ^18^F-FDG PET/CT, all PET/CTs showed distant metastases. Distant metastatic locations in the 23 patients included bone (15 patients), lungs (12 patients) and liver (11 patients). Surgical treatment included a total thyroidectomy with lymph node dissection in seven patients, hemithyroidectomy with lymph node dissection in two patients, palliative radiotherapy in four patients, TKI in five patients and watchful waiting or palliative care in the remaining five patients.

## Discussion

As the technique and quality of PET/CT imaging improves over time, knowing its value in the initial assessment of MTC patients could optimize stratification and treatment at diagnosis. In this two-center, retrospective study of 175 MTC patients, 35 ^18^F-FDG PET/CTs, 33 ^18^F-DOPA PET/CTs, 91 ultrasounds, 33 MRIs and 31 CTs were performed at diagnosis. Both PET/CT scans were superior to ultrasonography, CT and MRI in identifying lateral neck LNMs, with sensitivity rates of 89% and 81% and specificity rates of 100% and 100% for ^18^F-FDG PET/CT and ^18^F-DOPA PET/CT, respectively. For the central compartment LNMs, CT was superior, with a sensitivity of 93% compared to 72% on ^18^F-FDG PET/CT and 39% on ^18^F-DOPA PET/CT. While CT may therefore be the most optimal modality for identifying central LNMs, the high accuracy for lateral LNMs on preoperative PET/CT, as well as its ability to detect distant metastatic disease, may allow more optimal staging at diagnosis and de-escalation of the initial surgical strategy.

Literature on the role of PET/CT imaging in the preoperative process of MTC patients is very limited, which is highlighted by the international ATA and EANM guidelines that only give recommendations for PET/CT imaging in the postoperative phase [1, 9]. This is also demonstrated by the results of our study; only 35 ^18^F-FDG PET/CTs and 33 ^18^F-DOPA PET/CTs were made preoperatively. In addition, more PET/CTs were performed in the UMCG than in the UMCU, highlighting the variability in decisions between hospitals, when no guidelines are available for guidance and when variability in tracer availability exists. In contrast, current guidelines do recommend routine performance of ultrasound in evaluation of thyroid nodules to guide risk stratification. It is therefore quite surprising that only 91 patients had a preoperatively ultrasound. In recent years, ultrasound use has likely become more standard compared to the earlier years of the study period. In addition, the retrospective nature may have caused an underestimation of the total preoperative ultrasounds.

While some groups advocate for extensive LND to improve nodal staging and reduce locoregional recurrences, others suggest a more conservative LND at primary treatment to reduce morbidity, while risking another operation later on. Impact of either approach on survival remains contradictory [3, 14–17]. The second approach was likely utilized by the tertiary referral hospitals in this study, as evidenced by the fact that 33% of patients underwent LND, confirming LNMs in a significant proportion of patients (90%).

In our study, ^18^F-FDG PET/CT and ^18^F-DOPA PET/CT performed particularly well in the detection of LNMs in the lateral compartment, with sensitivity rates of 89% and 81% and specificity rates of 100% and 100%, respectively. For ^18^F-DOPA PET/CT, the sensitivity is somewhat better while the specificity is concordant with literature (sensitivity 73–75%, specificity 100% [7, 18]). Comparable data for ^18^F-FDG PET/CT are unavailable. Conventional imaging modalities seem less sensitive and specific than either PET/CT. Interestingly, even ultrasound is slightly inferior to either PET/CT, according to our study. Limited data on the sensitivity and specificity of ultrasound for lateral LNMs in MTC varies between 58–89% and 71–97% [7, 19]. With PET/CT, a more targeted LND may be possible, optimizing the surgical strategy after diagnosis. Given that patients with lateral LNMs typically have simultaneous central LNMs, one could argue that identifying lateral LNMs is particularly critical as it establishes the need for both CND and LND. In this retrospective study, none of the imaging modalities gave a sufficiently high sensitivity and negative predictive value to safely rule out affected lateral neck lymph nodes and subsequent LND.

The standardized approach to perform a CND in MTC guidelines is reflected in our data. LNMs were identified in 51% of 140 patients undergoing a CND, which is comparable to literature [20, 21]. The sensitivity for LNMs in the central compartment was 72% on ^18^F-FDG PET/CT, but relatively low on ^18^F-DOPA PET/CT (39%). The latter is however in line with the only other reported sensitivity rates for central compartment LNMs in the preoperative setting (28% and 53%) [7, 18]. The discrepancy between ^18^F-FDG PET/CT and ^18^F-DOPA PET/CT is difficult to explain but may be the result of selection bias, where the observation of aggressive clinical course led to the choice for ^18^F-FDG PET/CT. However, in general, the low sensitivity for central LNMS on PET/CT imaging may be explained by simultaneous tracer uptake in the primary thyroid lesion, influencing visualization of small lymphadenopathy in the preoperative setting [22]. Ultrasound seems impractical for the assessment of central LNMs, likely because their anatomic position is further from the surface. Diagnostic CT may be best at localizing LNMs in the central neck, and could even be combined with PET. In the future, intraoperative fluorescent imaging may provide advanced information on affected lymph nodes to optimize the extent of the CND, on an individual basis [23].

Since total thyroidectomy remains the recommended treatment for patients with locoregional MTC, the role of PET/CT in identifying thyroid tumor bilaterality may appear insignificant. However, some studies suggest that hemithyroidectomy may suffice in patients with sporadic, unilateral disease when intraoperative frozen section assessment reveals absence of desmoplasia [24–28]. In this study, ^18^F-FDG and ^18^F-DOPA uptake was confirmed as true positive in 71% and 58% of cases, respectively. False-negative rates were relatively high as well, likely due to partial-volume effects and low tracer uptake in smaller tumors [7]. Moreover, in bilateral tumors confirmed by histopathology, imaging may have correctly identified only one tumor, leading to its classification as false negative. The false-negative rate for ^18^F-DOPA PET/CT was higher than for ^18^F-FDG PET/CT, likely due to a larger proportion of hereditary cases, in whom bilateral tumors are more prevalent. While PET/CT may reinforce the decision to perform a total thyroidectomy when bilateral uptake is detected, the high false negatives rates indicate that PET/CT alone is insufficient to justify de-escalating treatment to hemithyroidectomy.

Due to the unpredictable behavior of MTCs, determining the optimal surgical treatment for an individual patient can be challenging [29]. Current guidelines recommend routine ultrasound assessment of the thyroid and lymph nodes but this advice is mostly based on its performance in other thyroid cancers. Ultrasound is relatively inexpensive and safe, and cannot be replaced by PET/CT completely due to its value in obtaining fine needle aspiration cytology. However, for accurate evaluation, an experienced radiologist is required to obtain reliable information, and images are difficult to review later on. In contrast, PET/CT provides reproducible, whole-body images showing metabolic activity and basic anatomic information. While PET/CTs may be relatively expensive, the superior specificity and positive predictive value mean initial surgery can be optimized with the addition of an LND, lowering the risk of reoperating in a later phase. Moreover, the capacity of PET/CT to show distant metastatic disease can limit unnecessary surgery when cure is impaired anyways. The lower surgical (recurrence) costs and associated impact on the environment, may off-set the balance in favor of preoperative PET/CT imaging. Finally, relatively high ^18^F-FDG avidity may provide valuable information on tumor biology and support more aggressive management [10, 30–32]. As such, future guidelines should consider to implement advice on performing preoperative PET/CT imaging to optimize staging and treatment at diagnosis. While expensive, the findings may ultimately contribute to achieving cost reductions in the long run.

While this study included quite a large number of patients for such a rare disease, several limitations should be reported. Real-life retrospective data were used to evaluate our clinical practice patterns; as a result, not all patients underwent standardized PET/CT imaging and bilateral LND, restricting sensitivity and specificity calculations to those who were operated on, and probably inducing selection bias. Additionally, PET tracer uptake in lymph nodes was analyzed at the neck compartment level rather than by individual cervical levels, limiting precise correlations between tracer uptake and the histopathological presence of LNM. Nonetheless, we believe this approach enhanced the reliability of the data in this setting, as retrospectively pinpointing exact locations from nuclear physicians’ clinical reports is both challenging and prone to error. Finally, the actual images were not reviewed which means that reported values may be underestimated. Ideally, a future study should prospectively evaluate the influence of preoperative ^18^F-FDG PET/CT, and ^18^F-DOPA PET/CT on the surgical strategy, in a randomized, controlled study design and should incorporate preoperative biochemistry and assess the impact on cost-effectiveness and surgical outcomes.

In conclusion, PET/CT imaging can offer valuable insights to guide and personalize the initial surgical strategy, particularly when combined with a diagnostic CT. While PET/CT may optimize the necessity to perform a lateral neck dissection, diagnostic CT seems most optimal for staging of the central compartment. The additional capacity of PET/CT to provide information on the presence of distant metastatic disease means surgical management can be de-escalated when cure is improbable anyways.

## Supplementary Information

Supplementary Material 1: Supplementary 1. True positive, false negative, true negative and false negative identification of central and lateral neck compartments on ^18^F-FDG PET/CT in relation with histopathology in the corresponding compartments.

Supplementary Material 2: Supplementary 2. True positive, false negative, true negative and false negative identification of central and lateral neck compartments on ^18^F-DOPA PET/CT in relation with histopathology in the corresponding compartments.

## Data Availability

The datasets used and analysed during the current study are available from the corresponding author on reasonable request.
