# Supplementary figures and images for: The value of 18F-FDG PET/CT and 18F-DOPA PET/CT in determining the initial surgical strategy of patients with medullary thyroid cancer: Preoperative PET/CT imaging for medullary thyroid cancer
Source: Cancer Imaging. 2025 Mar 26;25:41. doi: 10.1186/s40644-025-00862-4 (PMC11938770; doi:10.1186/s40644-025-00862-4)

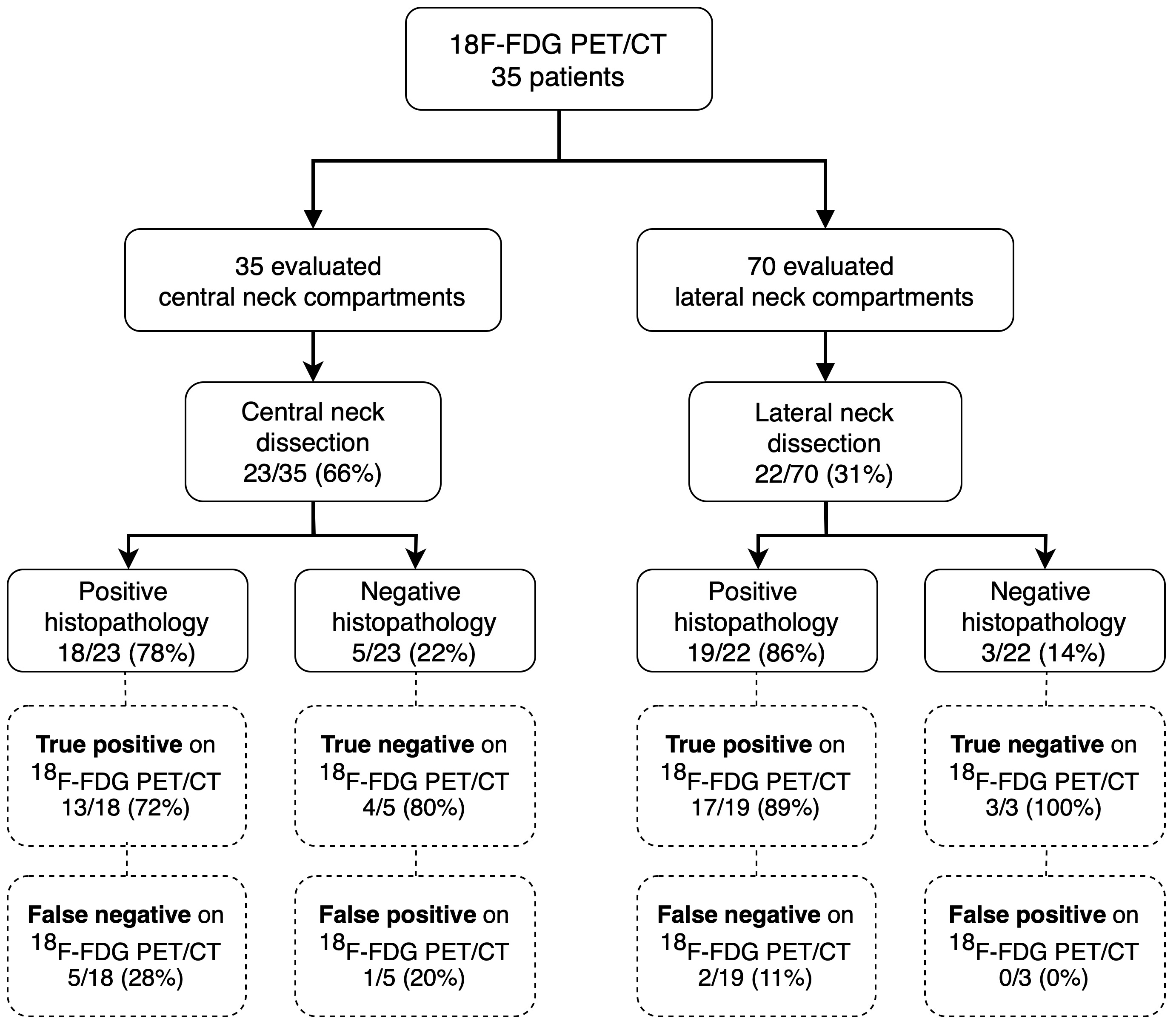

Supplement: Supplementary file 1 — Supplementary Material 1: Supplementary 1. True positive, false negative, true negative and false negative identification of central and lateral neck compartments on 18F-FDG PET/CT in relation with histopathology in the corresponding compartments. [file 40644_2025_862_MOESM1_ESM.jpg]

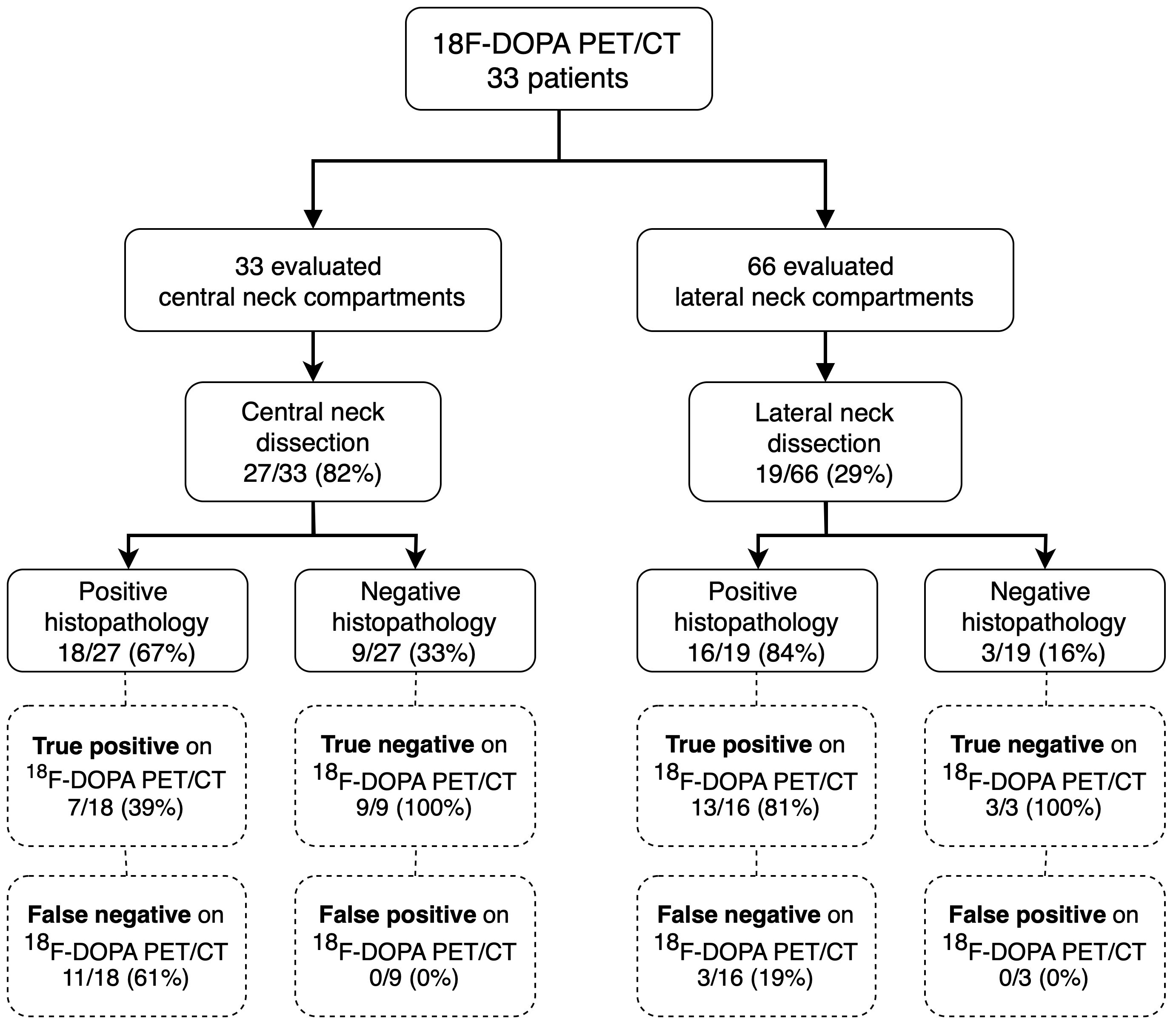

Supplement: Supplementary file 2 — Supplementary Material 2: Supplementary 2. True positive, false negative, true negative and false negative identification of central and lateral neck compartments on 18F-DOPA PET/CT in relation with histopathology in the corresponding compartments. [file 40644_2025_862_MOESM2_ESM.jpg]
